# Caring for Older Adults with Vision Impairment and Dementia: Data from the National Study of Caregiving

**DOI:** 10.1093/geroni/igaa057.841

**Published:** 2020-12-16

**Authors:** Varshini Varadaraj, Bonnielin Swenor, Shang-En Chyng, Orla Sheehan, Ashley Deemer, Joshua Ehrlich, Jennifer Wolff, David Roth

**Affiliations:** 1 Johns Hopkins University, Baltimore, Maryland, United States; 2 Johns Hopkins University School of Medicine, Baltimore, Maryland, United States; 3 University of Michigan, Ann Arbor, Michigan, United States; 4 Johns Hopkins Bloomberg School of Public Health, Baltimore, Maryland, United States

## Abstract

We examined caregiving relationships for individuals with vision impairment (VI) and dementia, using 2011 National Health and Aging Trends Study (NHATS) data, a survey of Medicare beneficiaries, linked to the National Study of Caregiving, a survey of family/unpaid helpers to NHATS participants. VI was defined as self-reported blindness or difficulty recognizing someone across the street, watching television or reading newspaper print. Dementia was defined as probable dementia based on survey-report or AD8 criteria. Caregiving outcomes included: (1) hours of care provided in the last month and (2) number of valued activities affected by caregiving. Among 1,196 caregivers, 617 assisted older adults without dementia or VI (D-/VI-), 298 with dementia but without VI (D+/VI-), 143 without dementia but with VI (D-/VI+), and 138 with dementia and VI (D+/VI+). In fully-adjusted regression models, caregivers of older adults D+/VI+ spent twice as many hours (IRR=2.0; 95%CI: 1.5-2.7) providing care than caregivers of older adults D-/VI-; however, caregivers of adults D+/VI- and those providing to older adults D-/VI+ spent 1.5-times more hours (95% CI=1.2-1.7; 95% CI=1.1-2.0, respectively). Additionally, caregivers of older adults D+/VI+ reported 4 times as many valued activities were affected (95%CI=2.8-5.6) then caregivers of those D-/VI-, while caregivers of those D+/VI- reported 1.9-times (95% CI=1.3-2.8) and D-/VI+ 1.6-times (95% CI=1.1-2.3) more activities were affected. Our results suggest that caring for older adults with VI has similar demands as caring for older adults with dementia, but that these implications may be magnified when caring for older adults with both dementia and VI.

